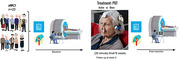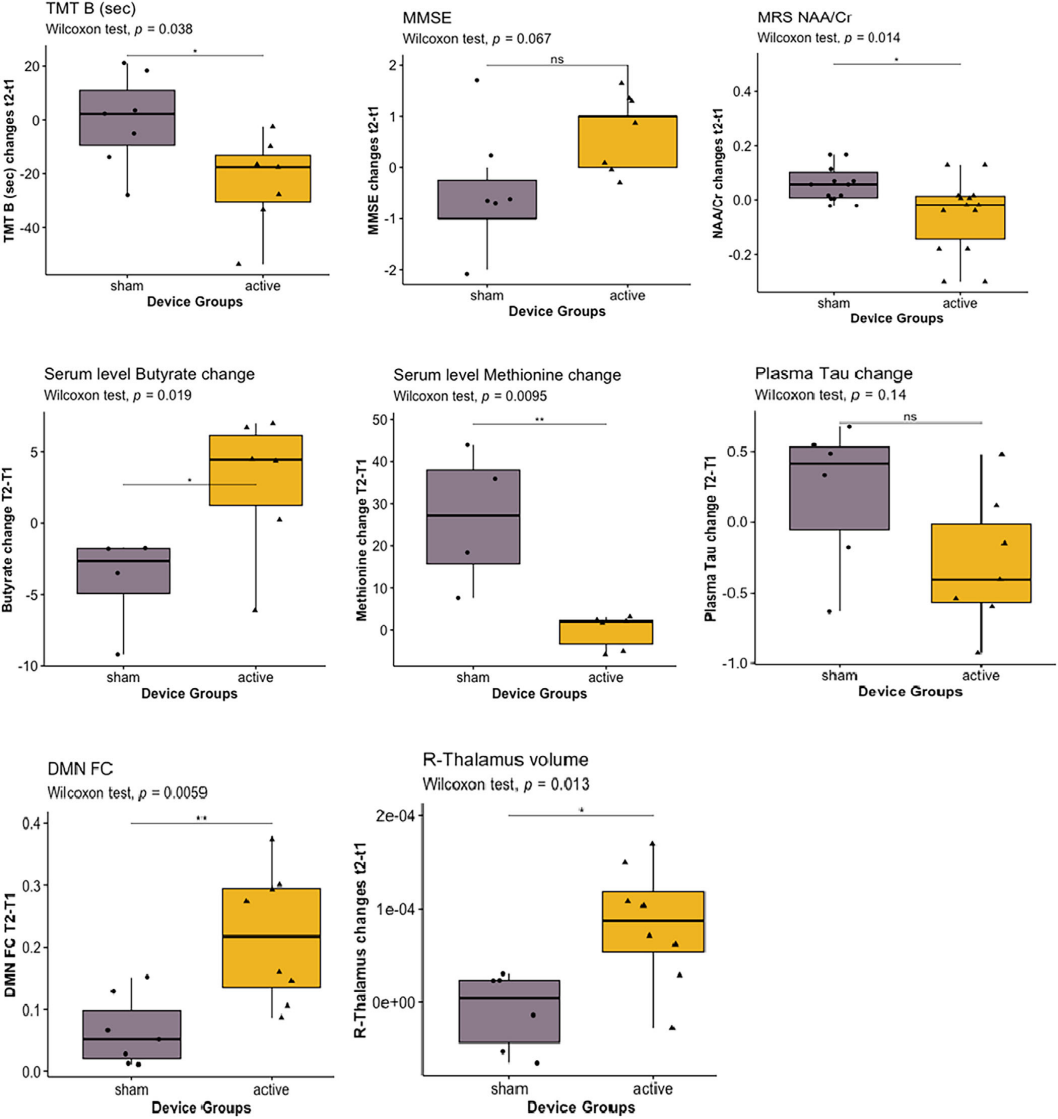# A pilot study evaluating the feasibility, safety, and efficacy of transcranial photobiomodulation (tPBM) for the treatment of mild cognitive impairment (MCI): preliminary findings

**DOI:** 10.1002/alz.095049

**Published:** 2025-01-09

**Authors:** Neda Rashidi‐Ranjbar, Nathan W. Churchill, Simon J. Graham, Raphael Schneider, Tarek K. Rajji, Ana C. Andreazza, Lew Lim, Jeff Coull, David G. Munoz, Luis R Fornazzari, Tom A. Schweizer, Corinne E. Fischer

**Affiliations:** ^1^ Keenan Research Centre for Biomedical Science, Li Ka Shing Knowledge Institute, St. Michael’s Hospital, Toronto, ON Canada; ^2^ Department of Medical Biophysics, University of Toronto, Toronto, ON Canada; ^3^ Sunnybrook Research Institute, Toronto, ON Canada; ^4^ Temerty Faculty of Medicine, Division of Neurology, University of Toronto, Toronto, ON Canada; ^5^ Campbell Family Mental Health Research Institute, Centre for Addiction and Mental Health, Toronto, ON Canada; ^6^ Toronto Dementia Research Alliance, University of Toronto, Toronto, ON Canada; ^7^ Temerty Faculty of Medicine, Department of Psychiatry, University of Toronto, Toronto, ON Canada; ^8^ Department of Pharmacology & Toxicology, Mitochondrial Innovation Initiative, University of Toronto, Toronto, ON Canada; ^9^ Vielight Inc., Toronto, ON Canada; ^10^ Weston Family Foundation, Hilary and Galen Weston Foundation, Toronto, ON Canada; ^11^ Temerty Faculty of Medicine, Department of Laboratory Medicine and Pathobiology, University of Toronto, Toronto, ON Canada; ^12^ Temerty Faculty of Medicine, Division of Neurosurgery, University of Toronto, Toronto, ON Canada

## Abstract

**Background:**

Mild Cognitive Impairment (MCI) is often a precursor to Alzheimer’s dementia (AD). Recent research underscores the relationship between mitochondrial dysfunction and amyloid‐beta accumulation, raising the prospect of targeting mitochondrial function for intervention. Transcranial photobiomodulation (tPBM), a non‐invasive technique utilizing near‐infrared light, has been shown to enhance mitochondrial function. Therefore, our study aimed to investigate the efficacy of tPBM in improving brain functions in MCI.

**Methods:**

Fourteen (N = 14) participants with MCI recruited at St. Michael’s Hospital Memory Disorders Clinic were randomized (1:1) to either active or sham tPBM (Neuro RX Gamma, Vielight Inc). Participants underwent daily home‐based active or sham tPBM sessions over 6 weeks. Pre‐ and post‐treatment assessments included a comprehensive battery of tests, encompassing the Trail Making Test (TMT B & A), Mini‐Mental State Examination (MMSE), Proton Magnetic Resonance Spectroscopy (H‐MRS) of the posterior cingulate cortex, structural MRI, resting‐state functional MRI (rsfMRI), and blood‐based biomarker was collected using NMR spectroscopy quantitative and Neurology 3‐Plex A (N3PA) Assays.

**Results:**

Comparing pre‐post changes in the active vs. sham group, we found significant differences favoring the active group (p’s<0.05) in: completing TMT‐B (t‐statistic = ‐2.1); for H‐MRS, decline in N‐acetyl aspartate to total creatine ratio (NAA/Cr) (t‐statistic = 2.8); increase in right thalamic volume (t‐statistic = 3.2); for rsfMRI, higher absolute change in functional connectivity in the default mode network (DMN) (t‐statistic = 3.5) and limbic network (t‐ statistic = 3.3), and between DMN and executive control network (ECN) (t‐statistic = 2.8); for blood results, decrease in isoleucine (t‐statistic = 3.0), methionine (t‐statistic = 3.1), and sarcosine (t‐statistic = 3.2) levels—markers linked to AD and amyloid plaque formation; and increase in butyrate (t‐statistic = ‐2.5) and L‐carnitine (t‐statistic = ‐2.6) levels—markers indicative of improved mitochondrial function. Furthermore, a notable trend was observed towards improved TMT B/A (p = 0.05, t‐statistic = ‐2.2) and MMSE (p = 0.06, t‐statistic = 2.0). Although the reduction in plasma tau levels within the active group was not statistically significant, it was notable (t‐statistic = 1.8).

**Conclusions:**

Our pilot study suggests that tPBM could improve executive function, neuronal health, functional connectivity, and mitochondrial function, and decrease AD markers in patients with MCI. Further research with larger sample sizes is essential to replicate and validate these results.